# Nutraceutical Profiles and FTIR Fingerprints of Comorian *Coffea canephora* and *Coffea liberica* var. *dewevrei*

**DOI:** 10.3390/metabo16050303

**Published:** 2026-04-29

**Authors:** Ahmed Irchad, Charaf Ed-dine Kassimi, Ibrahim Salmata, Hidaya Mansouri, Yssoufa Thabiti, Souhaila Hadday, Fayida Ahmed Mohamed, Rachid Aboutayeb, Hamza Abdou Azali, Cristèle Delsart, Lahcen Hssaini

**Affiliations:** 1 National Research Institute for Agriculture, Fisheries and Environment (INRAPE), Ex CEFADER, M’dé, Ngazidja, Moroni P.O. Box 1406, Comoros; irchadahmed04@gmail.com (A.I.); ibrahimsalmat61@gmail.com (I.S.); hidayam@nm-aist.ac.tz (H.M.); abdouazalihamza@gmail.com (H.A.A.); 2 Agro-Food Technology and Quality Laboratory, Regional Center of Agricultural Research of Meknes, National Institute of Agricultural Research, Rabat 10090, Morocco; charafeddine.kassimi@inra.ma (C.E.-d.K.); s.hadday@edu.umi.ac.ma (S.H.); 3 Laboratory of Science, Culture, Society and Development, University of Toamasina, door, CUR Barikadimy, Building B, 4th, Toamasina 501, Madagascar; thabityssouf@gmail.com; 4 Soil, Water, Plant Laboratory, Regional Center of Agricultural Research of Settat, National Institute of Agricultural Research, Avenue Ennasr, BP 415 Rabat Principale, Rabat 10090, Morocco; rachid.engineer@gmail.com; 5 École Supérieure d’Agro-Développement International (ISTOM), UR ADI-Suds (Agro-Développement et Innovation aux Suds), 4 rue Joseph Lakanal, 49000 Angers, France; c.delsart@istom.fr

**Keywords:** Comoros, *Coffea*, nutraceutical profiles, ionomic profile, vibrational spectroscopy

## Abstract

Background/Objectives: *Coffea canephora* (robusta) and *Coffea liberica* var. *dewevrei* (excelsa) cultivated in the Comoros islands represent understudied coffee varieties grown in a unique volcanic terroir. Despite their agricultural significance and potential bioactive value, no comprehensive biochemical or nutritional characterization of these Comorian coffees had previously been conducted. This study therefore aimed to provide the first integrated biochemical and nutritional characterization of both varieties and to evaluate the influence of the islands’ specific edaphoclimatic conditions on their chemical composition. Methods: An integrated analytical approach was employed, combining UV-Vis spectrophotometry, HPLC, ionomics, and FTIR-ATR spectroscopy to quantify polyphenols, flavonoids, caffeine, soluble sugars, antioxidant activity, mineral profiles, and macromolecular composition of green coffee beans from both species. Results: Robusta exhibited significantly higher levels of total polyphenols (121.79 ± 2.73 mg GAE/g), total flavonoids (29.43 ± 2.20 mg QE/g), caffeine (1.52% *w*/*w*), total soluble sugars (60.47 ± 3.37 mg GE/g), and antioxidant activity (64.97 ± 6.25 mM Trolox eq/g). Conversely, excelsa demonstrated a distinct mineral profile, with significantly higher concentrations of magnesium, calcium, sodium, zinc, and copper. FTIR spectroscopy confirmed distinct vibrational fingerprints between the two species, particularly in lipid and carbohydrate signatures. Conclusions: These findings position Comorian robusta as a potent source of antioxidants and stimulants, while excelsa offers a nutritionally balanced profile with nutraceutical potential, providing a scientific basis for valorizing both varieties as high-value niche products and contributing to the preservation of coffee agro-biodiversity.

## 1. Introduction

Coffee is one of the most widely consumed beverages globally and serve as a major agricultural commodity, renowned for its diverse health benefits. The global coffee market is predominantly dominated by two species: *Coffea arabica* (arabica) and *Coffea canephora* (robusta). In addition to these primary species, a third species, *Coffea liberica* var. *dewevrei* (commonly known as excelsa), remains largely understudied despite its agronomic value. Excelsa exhibits remarkable resistance to drought and certain pathogens, notably the devastating fungus *Hemileia vastatrix* (causal agent of coffee leaf rust) [1]. This large-fruited coffee (excelsa) is often extensively cultivated, and considered as a rare, mysterious, and even elusive commercial coffee variety [2,3,4]. This status is partly attributable to its limited contribution of approximately 1% to global production, whereas arabica (*Coffea arabica* L.) (55%) and robusta (*Coffea canephora* Pierre ex A. Froehner) (45%) remain the two most widely cultivated and traded varieties [5]. The limited global success of excelsa at the beginning of the twentieth century has been largely attributed to several factors, including the inappropriate selection of plant material for its worldwide dissemination. However, a renewed interest in this species is now evident throughout the coffee supply chain. Nevertheless, the vast biochemical diversity within these species, which is heavily influenced by terroir, including the unique interaction between genotype, soil, climate, and agricultural practices remains largely unexplored for many lesser-known geographical origins.

The Comoros Archipelago, a group of four main volcanic islands (Grande Comore (Ngazidja), Moheli (Mwali), Anjouan (Ndzuwani), and Mayotte (Maore)) located in the Mozambique Channel northwest of Madagascar, represents one such understudied terroir. Ngazidja (Grand Comore) is the youngest and largest island of the archipelago, with an active volcano (Karthala). These islands host numerous plant species and fruits specific to the tropical climate and environment, some of which are endemic. The Comorian flora exhibits biogeographic affinities with that of Malagasy and East African flora, and to a lesser extent, that of the Seychelles [6]. The volcanic microclimate of the Comoros Archipelago, its mineral-rich basaltic soils, and its biogeographic isolation create a distinctive environment for coffee cultivation. Once a historical producer, the region has experienced a significant decline in production, raising concerns over the potential loss of a potentially exceptional genetic and biochemical resource. While coffees from renowned terroirs in Ethiopia, Brazil, or Yemen have been the subject of numerous metabolomic studies, coffee plants cultivated under the distinctive pedoclimatic conditions of the Comoros Archipelago have, to date, not benefited from any thorough scientific investigation. This knowledge gap not only impedes the valorization of Comorian coffee but also hinders a comprehensive understanding of the metabolic diversity within the *Coffea* genus. Yet, this so-called “stimulating” beverage represents one of the world’s attractive and profitable agro-industrial sectors, largely due to its positive health effects [7,8]. Moderate coffee consumption is associated with beneficial effects, reducing the incidence of several chronic disorders, including diabetes [9], hypertension, as well as Alzheimer’s and Parkinson’s diseases [10,11,12,13]. Positive effects have also been reported on hepatic and renal diseases [14,15,16,17,18]. Furthermore, the identification of polyphenols and antioxidants in coffee has further increased its appeal [19,20,21].

From a biochemical perspective, the coffee bean is one of the most complex food products. Its most widely reported constituents are carbohydrates, minerals, caffeine, chlorogenic acid, amino acids, and lipids. However, their concentrations can vary significantly depending on the variety, cultivation conditions, climate, terroir, harvest, and post-harvest processing [22,23,24,25]. Gonzalez-Rios et al. [26] reported that cherries processed by the wet method exhibit superior quality compared to those processed by the dry method, and that the type of pulping influences the coffee’s flavor profile. Microbial pulping, in particular, yields coffee with enhanced aromatic quality. Furthermore, studies have established a correlation between the geographical origin of coffee and its aromatic composition [27], its chemical composition [28], and the polymorphism of its genetic bases [29]. Indeed, the chemical profile of coffee enables the differentiation of varieties, the identification of geographical origins, the discrimination of production processes, and the assessment of nutritional quality [30]. Nutrient content reflects local environmental conditions, such as climate and soil composition, as well as the application of fertilizers and pesticides [31,32]. Additionally, coffee serves as a significant source of major and trace elements, including K, Ca, Na, Mg, Fe, Zn, Cu and Mn [13].

As coffee-producing nations seek to develop local production and capture greater added value, it is hypothesize that the unique edaphoclimatic conditions of the Comoros archipelago confer a distinctive metabolic signature to the cultivated robusta and excelsa beans. These conditions are expected to generate different profiles of biofunctional metabolites and nutritional components including lipids, proteins, sugars, and micronutrients compared with beans of the same species grown elsewhere. Consequently, this study aims to conduct the first comparative untargeted metabolomic analysis and comprehensive nutritional profiling of *C. canephora* (robusta) and *C. liberica* var. *dewevrei* (excelsa) grown in the local conditions of the Comoros Archipelago to elucidate their nutritional, antioxidant, and agro-industrial potential. The analytical approach combines advanced techniques: classical biochemical analyses (UV-Vis spectrophotometric, High-Performance Liquid Chromatography), ionomics, and vibrational fingerprinting using Fourier Transform Infrared spectroscopy with Attenuated Total Reflectance (FTIR-ATR). This integrated methodology is designed to enhance the robustness and relevance of the findings, thereby providing a more comprehensive basis for interpretation. This innovative study and holistic approach will establish a robust scientific foundation to position Comorian robusta and excelsa coffees as high-value niche products, rich in health-beneficial compounds. Furthermore, it will contribute to the preservation of this neglected agro-biodiversity.

## 2. Materials and Methods

### 2.1. Sample Preparation

Coffee cherries (*Coffea canephora* var. *robusta* and *Coffea liberica* var. *dewevrei*) were harvested in July 2022 from a pesticide-free plantation in Mkazi (Bambao region, Ngazidja Island, Comoros; 11° 48′ 39″ S, 43° 16′ 01″ E). Fruits at full maturity were randomly collected from trees, defined by a red-orange exocarp coloration covering approximately three-quarters of the fruit and easy detachment from the pedicel. Sampling was performed from various locations within the tree canopy. After harvest, the cherries were manually depulped and processed using the wet method as described by Hamdouche [23]. The separated seeds were thoroughly washed with distilled water and air-dried at ambient temperature for 72 h. Subsequently, the dried beans were ground into a fine powder using an IKA A11 basic analytical grinder (IKA-Werke GmbH & Co. KG, St. Louis, MO, USA) at room temperature. The resulting powders were stored at −18 °C in airtight containers pending further analysis to prevent degradation.

### 2.2. UV-Vis Spectrophotometric Analysis

#### 2.2.1. Hydroethanolic Preparation for Phenolic, Sugar, Tannin, and Antioxidant Analyses

The ethanol extraction was performed based on the method described by Sanders et al. [33], with modifications introduced by Xie and Bolling [34]. Briefly, 1 g of dried coffee bean powder was homogenized in 20 mL of a hydroethanolic solution (ethanol/ultrapure water, 80:20, *v*/*v*) using an IKA T-18 Basic Ultra-Turrax homogenizer (IKA Werke GmbH & Co., Staufen, Germany). Homogenization was carried out at 4 °C for 15 min. The resulting homogenate was subsequently centrifuged at 3000× *g* for 10 min at 4 °C. The supernatant was collected, and the residual pellet was subjected to two additional cycles of homogenization and centrifugation under identical conditions. The combined supernatants from all three extractions were finally filtered through Whatman No. 1 filter paper.

#### 2.2.2. Determination of Total Phenolic Content

The total phenolic content (TP) was quantified according to the Folin–Ciocalteu microassay method [35]. The reaction mixture was prepared by combining 40 μL of the extract, 3160 μL of ultrapure water, 200 μL of Folin–Ciocalteu reagent, and 600 μL of a 20% (*w*/*v*) sodium carbonate (Na_2_CO_3_) solution. Following incubation at 40 °C for 30 min, the absorbance was measured at 765 nm. The TP was calculated based on a gallic acid standard curve and is expressed as milligrams of gallic acid equivalent per gram of dry weight (mg GAE/g DW).

#### 2.2.3. Determination of Total Flavonoid Content

The total flavonoid content (TF) was determined according to the aluminum chloride colorimetric method described by Barreira et al. [36], with modifications. Briefly, 250 µL of the extract was mixed with 1.25 mL of ultrapure water and 75 µL of a 15% (*w*/*v*) sodium nitrite (NaNO_2_) solution. After 5 min of incubation, 150 µL of a 10% (*w*/*v*) aluminum chloride (AlCl_3_) solution was added. Six minutes later, the reaction was stopped by adding 500 µL of 1 M sodium hydroxide (NaOH) and 275 µL of ultrapure water. The solution was vortexed thoroughly, and the absorbance of the resulting pink complex was measured at 510 nm using an appropriate blank. The TF was quantified based on a quercetin standard curve and expressed as milligrams of quercetin equivalent per 100 g of dry weight (mg QE/100 g DW).

#### 2.2.4. Determination of Total Soluble Sugars

Total soluble sugars (TSS) were quantified colorimetrically according to the phenol-sulfuric acid method [37], using glucose as a standard. Briefly, 100 µL of extract was combined with 500 µL of 5% (*w*/*v*) aqueous phenol and 2.5 mL of concentrated (96%) sulfuric acid. The reaction mixture was incubated for 10 min at room temperature, followed by a 12 min incubation in a water bath maintained at 30 °C. Absorbance was measured at 490 nm. TSS content was expressed as milligrams of glucose equivalents per 100 g of dry weight (mg GE/100 g DW).

#### 2.2.5. Determination of Hydrolysable Tannins

Hydrolysable tannin (HTs) content was quantified according to the spectrophotometric method of Willis [38], using tannic acid as an external standard. Briefly, 1 mL of the sample extract was mixed with 5 mL of a 2.5% (*w*/*v*) potassium iodate (KIO_3_) solution and vortexed for 10 s. The absorbance of the resulting mixture was measured at 550 nm. HT concentrations were determined from a standard curve and are expressed as milligrams of tannic acid equivalent per 100 g of dry coffee matter (mg TAE/100 g DW).

#### 2.2.6. Antioxidant Activity Assay

The free radical-scavenging capacity of the extracts was determined using the 2,2-diphenyl-1-picrylhydrazyl (DPPH) assay, following the method of Kumar et al. [39] with minor modifications. This assay only evaluates the in vitro antiradical activity of lipophilic extracts. Briefly, 0.2 mL of each extract was mixed with 3.8 mL of a freshly prepared DPPH solution (0.026 mg/mL in 96% ethanol). The reaction mixture was incubated in the dark for 30 min at room temperature, after which the absorbance was measured at 515 nm. The antioxidant capacity was calculated according to Equation (1).

I (%) = [(Abs control − Abs sample)/Abs control] × 100
(1)

The results were expressed as millimoles of Trolox equivalent per 100 g of dry weight (mmol Trolox eq/100 g DW), calculated using Equation (2).

(2) $$\mathrm{mM}\mathrm{trolox}\mathrm{eq}=\frac{(({I\%}_{sample}-b)/a)(mg/mL)\times 10^{3}}{c_{sample}(mg/mL)\times M_{Trolox}(g/mol)}$$

where

Abs: Absorbance; “a” and “b” are the slope and the y-intercept, respectively, of the linear regression line from the standard curve.

M_Trolox_: Molar mass of trolox;

C_Sample_: Sample concentration.

UV-Vis spectroscopic measurements were carried out in triplicate using a Shimadzu UV-1800 spectrophotometer (Kyoto, Japan).

### 2.3. High-Performance Liquid Chromatography (HPLC) Analysis

#### 2.3.1. Extraction Procedure

Both species (*C. canephora* (robusta) and *C. liberica var. dewevrei* (excelsa)) were analyzed in triplicate. The extraction was specifically carried out for the determination of caffeine. Ultrapure water was used as the extraction solvent. All solvents used for chromatographic analysis were of HPLC grade, while those used for extraction were of analytical grade (>99% purity). Ultrapure water was produced, filtered, and used throughout the experiments.

The extraction procedure was performed according to the method described by Belguidoum et al. [40], with modifications adapted from Lemos et al. [41]. Briefly, 7 mg of each coffee powder sample was extracted with 5 mL of ultrapure water at 95 °C for 5 min. Subsequently, 2.5 mL of methanol was added, and a second extraction was carried out using an ultrasonic water bath (80 kHz, 37 °C) for 20 min. The mixture was then centrifuged at 2136× *g* for 3 min. An additional 2.5 mL of methanol was added to the supernatant to obtain a final concentration of 0.7 mg/mL. The combined supernatants were collected for analysis. All extractions were performed in triplicate to ensure analytical reliability.

#### 2.3.2. Determination of Caffeine

Caffeine quantification was performed by high-performance liquid chromatography (HPLC) according to a previously established method [42], with modifications. Prior to analysis, coffee extracts were filtered through a 0.45 μm membrane. Caffeine quantification was performed using an external calibration curve with a caffeine standard (Sigma-Aldrich, St. Louis, MO, USA).Separations were conducted on an HPLC system (Breeze, Waters, Milford, MA, USA) equipped with a C18 reversed-phase column (150 × 4.6 mm, 5 μm; GL Sciences, Tokyo, Japan) and a UV detector (Waters 2489, Milford, MA, USA). Detection was performed at a wavelength of 274 nm. The mobile phase, consisting of 30% ethanol and 0.1% (*v*/*v*) acetic acid in water, was delivered isocratically at a flow rate of 1.4 mL/min. The total run time per analysis was 12 min.

### 2.4. Determination of Protein Content

Protein quantification was performed on coffee flour produced by grinding the seeds. Protein content was determined based on nitrogen (N) content using the Kjeldahl method (Büchi, Switzerland). Briefly, 0.25 g of dry coffee powder was digested with 6 mL of concentrated sulfuric acid in a heating block at 420 °C for 1.5 h. The digested sample was then distilled, and the liberated ammonia was titrated with standardized 0.05 N sulfuric acid. Protein content was calculated from the nitrogen content using a conversion factor.

### 2.5. Analysis of Mineral Composition

A mineral analysis of elements (Fe, Zn, Cu, Mn, K, Ca, Na and Mg) was conducted on coffee flour using a dry ashing method. Approximately 1.0 g of coffee flour was weighed into a porcelain crucible and incinerated in a muffle furnace. The temperature was gradually raised to 550 °C where it was maintained for 5 h. The resulting ash was cooled to room temperature. For the analysis of microelements (Fe, Zn, Cu and Mn), the cooled ash was dissolved in 5 mL of 2 N hydrochloric acid. After 15–20 min, the solution was diluted to a final volume of 50 mL with distilled water. The concentrations of these microelements were determined using an atomic absorption spectrometer (AAS) (PerkinElmer, Norwalk, CT, USA) [43]. Macroelements (K, Ca, Na and Mg) were extracted from the ash using nitric acid (HNO_3_) and quantified using a flame photometer (Model CL 378, Nanolytik, Germany) [44]. All analyses were performed in triplicate.

### 2.6. Fourier Transform Infrared (FTIR) Spectroscopy

Fourier transform infrared (FTIR) spectra of coffee seeds were acquired using a Perkin-Elmer spectrometer (Perkin Elmer, Waltham, MA, USA) equipped with a germanium attenuated total reflectance (ATR) crystal. The spectra were collected over the range of 4000 to 450 cm^−1^ at a resolution of 4 cm^−1^, with each spectrum representing the average of 128 accumulated scans to enhance the signal-to-noise ratio. Prior to sample analysis, a background spectrum of the clean crystal surface was collected and automatically subtracted from all subsequent sample spectra. For analysis, a constant sample mass of 50 mg was used. A maximum pressure of 1700 kg/cm^2^ was applied to ensure uniform contact between the sample and the crystal, which is critical for obtaining high-quality spectra [45]. The germanium crystal was cleaned between samples with ethyl alcohol and warm water, followed by careful drying with absorbent paper. The acquired spectra were processed using Essential FTIR software (version 3.50.183). The raw data were first corrected using the embedded ATR correction algorithm to account for the depth of penetration of the evanescent wave (incidence angle = 45°; number of ATR reflections = 1; sample refractive index = 1.5). This evanescent field is generated when infrared light reflects at the interface between the high-refractive-index crystal and the lower-refractive-index sample [46]. Subsequently, a baseline correction was applied, followed by standard normal variate (SNV) and multiplicative scattering correction (MSC) treatments to minimize the effects of light scattering and multiplicative interferences [47]. The processed spectra of the coffee beans revealed distinct fingerprint regions. The integrated intensities of these spectral features were quantified using the Essential FTIR software.

### 2.7. Statistical Analysis

All analyses were performed in triplicate, and data are presented as mean ± standard deviation. Prior to statistical analysis, data were assessed for normality (Shapiro–Wilk test) and homogeneity of variance (Levene’s test). Significant differences between means were determined using one-way analysis of variance (ANOVA) followed by Tukey’s honestly significant difference (HSD) post hoc test for parametric data. For non-parametric data, the Kruskal–Wallis test was applied. An independent samples *t*-test was used for comparisons between two groups. The Duncan’s multiple range test (DMRT) was also employed for mean separation. All statistical analyses were performed at a 95% confidence interval (*p* < 0.05) using SPSS software (version 23; IBM Corporation, Armonk, NY, USA).

## 3. Results

### 3.1. Total Phenolics, Total Flavonoids, Soluble Sugars, Tannins, Antioxidant Activity & Caffeine

**Figure 1:**
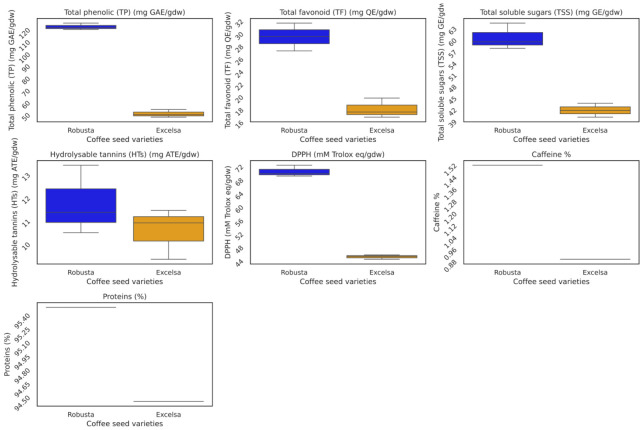
Comparison of biofunctional metabolites, nutritional components (proteins), and DPPH radical scavenging activity in robusta and excelsa beans from the Comoros.

The comparative analysis of biofunctional metabolites and DPPH radical scavenging activity in robusta and excelsa beans cultivated in the Comoros revealed significant interspecies differences for the majority of parameters evaluated. Total polyphenol and total flavonoid contents were significantly higher in robusta (121.79 ± 2.73 mg GAE/g and 29.43 ± 2.20 mg QE/g, respectively) than in excelsa (49.08 ± 4.25 mg GAE/g and 15.98 ± 3.26 mg QE/g) (Table 1, Figure 1). Antioxidant activity assessed by the DPPH assay was also significantly higher in robusta (64.97 ± 6.25 mM Trolox eq/g) compared to excelsa (41.74 ± 5.29 mM Trolox eq/g) (Table 1). No significant difference was observed for hydrolysable tannins between the two species (F = 0.208; *p* = 0.672) (Table 1). Caffeine content was significantly higher in robusta (1.52%) compared to excelsa (0.89%) (F = 1.58 × 10^37^; *p* = 2.41 × 10^−56^) (Table 1). Total soluble sugars (TSS) were significantly more abundant in robusta (60.47 ± 3.37 mg GE/g) than in excelsa (41.09 ± 2.84 mg GE/g) (Table 1).

### 3.2. Nutritional and Mineral Composition

Protein content was slightly but significantly higher in robusta (95.48%) compared to excelsa (94.45%), with a corresponding difference in total nitrogen content (1.86% vs. 1.61%, respectively) (Table 1, Figure 1).

**Figure 2:**
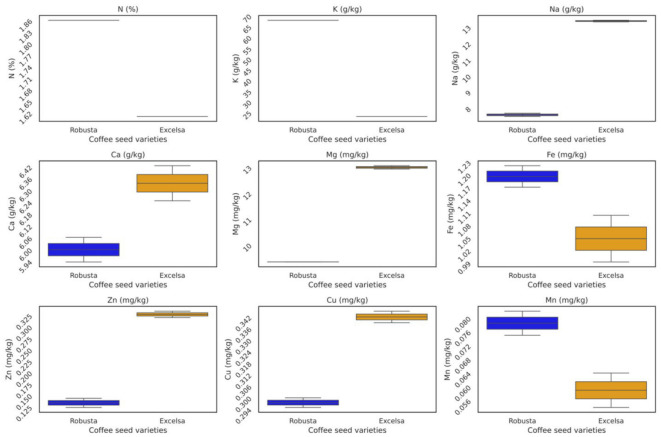
Distinctive mineral signature of robusta and excelsa coffee beans from the Comoros.

The mineral composition revealed marked contrasts between the two species (Table 1*).* (Robusta) exhibited significantly higher concentrations of potassium (K: 67.90 g/kg), manganese (Mn: 0.079 mg/kg), and iron (Fe: 1.20 mg/kg). Conversely, *C. dewevrei* (excelsa) was characterized by significantly higher levels of magnesium (Mg: 12.99 g/kg), calcium (Ca: 6.34 g/kg), sodium (Na: 13.35 g/kg), zinc (Zn: 0.33 mg/kg), and copper (Cu: 0.34 mg/kg) (Table 1, Figure 2).

### 3.3. FTIR Spectroscopic Profiles

**Figure 3:**
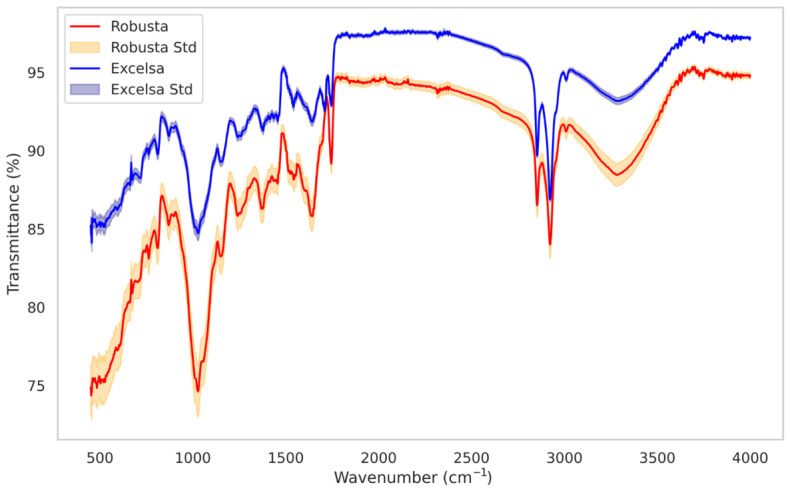
FTIR spectra (mid-infrared region, 4000–450 cm^−1^) of robusta and excelsa beans from the Comoros.

**Figure 4:**
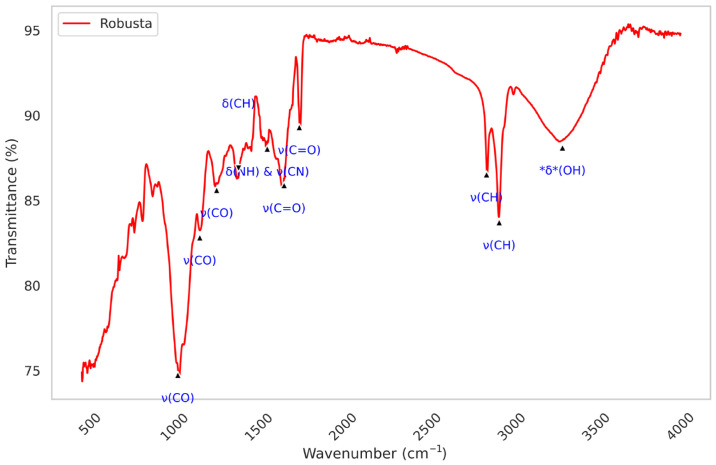
Representative FTIR spectrum of robusta samples in the 4000–450 cm^−1^ region.

FTIR-ATR spectroscopy was used to determine the vibrational profiles of robusta and excelsa from the Comoros. Figure 3 displays the spectra of the studied coffee samples across the 4000–450 cm^−1^ region. Characteristic peaks associated with major constituents such as caffeine, carbohydrates, water, and proteins were identified. As expected, the spectra of the studied samples are predominantly similar and overlapping, revealing significant structural commonalities. Figure 4 displays a representative IR spectrum of robusta samples, with the principal absorption bands labeled.

**Figure 5:**
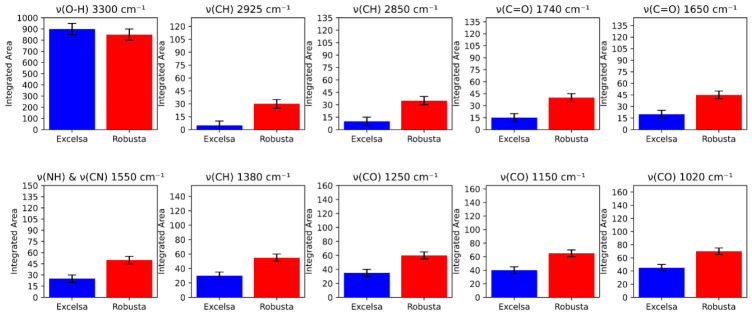
Integrated intensities of the main vibration regions calculated for the studied coffees.

Comprehensive spectral analyses were conducted at the following distinct major frequencies: ν(O–H) 3300 cm^−1^, ν(C-H) 2925 cm^−1^, ν(C-H) 2850 cm^−1^, ν(C=O) 1740 cm^−1^, ν(C=O) 1650 cm^−1^, ν(N-H) & ν(C-N) 1550 cm^−1^, ν(C-H) 1380 cm^−1^, ν(C-O) 1250 cm^−1^, ν(C-O) 1150 cm^−1^, and ν(C-O) 1020 cm^−1^ (Figure 5). Each spectrum represents the average of ten replicates, each corresponding to an accumulation of 128 scans. The integrated intensities across all major vibrational frequencies were calculated using OriginLab Pro v9 software and plotted (Figure 5). A one-way ANOVA revealed statistically significant differences (*p* < 0.05) in the integrated intensity at each major absorption frequency between the two species.

## 4. Discussion

### 4.1. Phytochemical Distinctiveness of Comorian Robusta and Excelsa

The higher polyphenol content observed in robusta is consistent with findings reported by Farah and Donangelo [48] and Ludwig et al. [49], who consistently documented greater concentrations of total phenolics in robusta relative to other species. Typical values for green robusta coffee determined by the Folin–Ciocalteu method generally range between 50 and 110 mg GAE/g DW [49,50], compared to 30–60 mg GAE/g DW for arabica. However, the values measured in the present study exceed those reported for robusta from Brazil (18–25 mg GAE/g) [51] or Vietnam (80–100 mg QE/g) [52], highlighting the biochemical distinctiveness of the Comorian terroir. This elevated polyphenol accumulation can be partly explained by the tetraploid nature of robusta, which sustains a more active secondary metabolism leading to greater accumulation of total phenolics [53]. Beyond genetic determinism, the volcanic and high-irradiance conditions of the Comoros likely stimulate the biosynthesis of secondary metabolites [54,55]. The high antioxidant activity measured in robusta is strongly correlated with its polyphenol content [49] and surpasses values typically reported for robusta from Ethiopia or Uganda [56], further supporting the role of the island’s edaphoclimatic conditions. However, the DPPH assay used in this study specifically measures the antiradical activity of compounds soluble in organic solvents, whereas the ABTS radical cation decolorization assay, employed in other studies, evaluates both hydrophilic and lipophilic antioxidants. Plant extracts are complex multicomponent matrices containing thousands of distinct compounds. Consequently, the scientific literature recommends using at least two complementary methods for reliable in vitro antioxidant assessment. To fully characterize the in vitro antioxidant activity of the tested extracts, additional assays are therefore required. Thus, the inclusion of complementary tests (e.g., ORAC, FRAP, or CUPRAC) represents a key perspective for future research.

The absence of a significant difference in hydrolysable tannins between the two species suggests that this parameter is less species-dependent and may be more influenced by external factors such as ripening stage or post-harvest practices [57]. Although the work of Campa et al. [58] established that robusta accumulates significantly higher concentrations of total phenolics including chlorogenic acids and their precursors than arabica, it did not specifically quantify hydrolysable tannins, identifying robusta as having a globally more active phenolic metabolism overall.

The higher caffeine content in robusta (1.52%) is consistent with the expected genetic profile of the species [58] and falls within the upper range of typical values (1.7–3.5%) reported by Farah and Donangelo [48], potentially reflecting a response to abiotic stresses induced by the island’s environmental conditions [55]. The scientific literature has consistently demonstrated significant interspecies differences in caffeine content; a comparative study revealed an average caffeine content of 2.26% for robusta versus 1.49% for arabica [51]. The intermediate caffeine level in excelsa (0.89%) aligns with published data indicating that excelsa generally exhibits caffeine contents comparable to or lower than arabica, with significant intra-specific variability attributed to genotype, terroir, and post-harvest practices [59].

The higher TSS content observed in robusta contrasts with several studies on robusta by Geromel et al. [60] reporting lower sugar contents relative to arabica, a difference considered a major determinant of robusta’s more pronounced bitterness and less sweet cup profile. The work of Mondolot et al. [61] on coffee bean cell walls suggests that the composition and structure of cell wall polysaccharides, closely linked to the TSS pool, vary considerably among species, directly affecting the bioavailability of aroma precursors during roasting. A synthesis by Leroy et al. [62] corroborates these findings, emphasizing that genotype is a primary factor explaining differences in green bean composition, including sugar content. The scarcity of published data on excelsa TSS profiles further underscores the novelty of these findings and necessitates tailored post-harvest protocols to optimize their sugar profile.

### 4.2. Nutritional and Mineral Fingerprinting of Comorian Coffea Robusta and Excelsa

The slightly higher protein and nitrogen contents in robusta may reflect both genotypic characteristics and favorable nitrogen assimilation conditions in the Comoros, consistent with the documented influence of pedoclimatic and agronomic factors on final grain composition [59,63,64]. The distinct mineral profiles between the two species are consistent with their genetic and physiological characteristics [65]. The notably elevated Zn and Cu concentrations in excelsa are particularly remarkable and may be associated with the volcanic nature of Comorian soils, which are naturally rich in trace elements [66]. The distinct profiles highlighted further underscore the influence of geographic origin and pedoclimatic factors on the biochemical differentiation of coffees [67]. From a nutraceutical perspective, excelsa’s richer profile in essential minerals (Mg, Ca, Na, Zn, Cu) positions it as a candidate of nutritional interest [68], while its uniqueness warrants valorization through geographical indications or quality labels [3,69]. Robusta, with its elevated antioxidant and stimulant profile, is conversely positioned as an exceptional source of natural antioxidants and biofunctional metabolites. Further studies incorporating sensory and genomic analyses, along with detailed characterization of chlorogenic acids and volatile compounds, are required to consolidate these findings and promote Comorian terroir coffees on the international market.

### 4.3. FTIR Spectroscopic Interpretation

The obtained FTIR spectra exhibit characteristic absorption bands corresponding to the major constituents of coffee, consistent with previous literature [70,71,72], and are in agreement with reports from several studies on coffee [73,74,75,76,77]. The detailed assignment of major vibrational bands identified in the spectra of robusta and excelsa is presented in Table 2.

In the 3600–3000 cm^−1^ region, the broad band centered at approximately 3300 cm^−1^ is attributed to O–H stretching vibrations from sugar alcohol hydroxyl groups, carboxylic acids, and phenolic acids (including chlorogenic acid), or to residual water, and may also correspond to N–H stretching of amines and amides from amino acids and proteins [80,81,82]. The weak band at ~3070 cm^−1^, characteristic of the (=C–H) stretching vibration, is likely associated with fatty acid unsaturation [95].

In the 3000–2800 cm^−1^ region, the asymmetric (ν_as_) and symmetric (ν_s_) stretching vibrations of CH_2_ and CH_3_ groups at approximately 2925 cm^−1^ and 2850 cm^−1^ are ascribed to aliphatic chains of lipids and to C–H bonds in caffeine molecules [70,71,83,84]. The slightly greater intensity of these bands in robusta suggests a distinct lipid content and composition, potentially associated with a higher level of cafestol and kahweol diterpenoid compounds with significant biological effects [96]. The intense peak at ~1740 cm^−1^, assigned to the carbonyl ν(C=O) stretching vibration of esters (primarily triglycerides), is more pronounced in robusta, further suggesting a higher lipid content [83,85]. According to Pavia et al. [85], this region is close to the 1725–1700 cm^−1^ region attributed to carbonyl stretching of carboxylic acids and ketones which are known to contribute to coffee acidity [74,97] and to aroma notes such as woody, cucumber, and almond [98].

The band at ~1650 cm^−1^, attributable to amide I (proteins) and caffeine vibrations, exhibits a spectral shift between the two species (1654 cm^−1^ vs. 1648 cm^−1^). This may reflect conformational differences in proteins or relative caffeine contents, consistent with comparative studies of robusta and *C. arabica* [78]. The position and intensity of this amide I band constitute a key indicator of protein content, consistent with the biochemical data showing higher protein levels in robusta. The 1650–1600 cm^−1^ region also receives contributions from caffeine [86,87,88], trigonelline [99], and hydrogen bonding of adsorbed water, free fatty acids, and chlorogenic acids [95].

In the 1600–1500 cm^−1^ region, the band at approximately 1550 cm^−1^ is primarily attributed to N–H bending vibration of amide II coupled with C–N stretching vibrations of proteins [89,90,91]. The fingerprint region (1500–900 cm^−1^) is particularly complex, arising from bending and stretching vibrations of various biomolecular bonds [100]. The band at ~1250 cm^−1^ is characteristic of C–O stretching vibrations of polysaccharides (cellulose, hemicelluloses) and asymmetric C–O–C stretching of glycosidic linkages [90], reflecting the high fiber content of the coffee bean matrix [101,102,103,104]. The spectral region between 1450 and 1150 cm^−1^ has been identified as characteristic of chlorogenic acids by several authors [70,71,78,79,85,86,88,93]. The C–O axial deformation of the quinic acid moiety occurs at approximately ~1020 cm^−1^, while the angular deformation is observed at ~1380 cm^−1^ [70,71], although these bands have alternatively been assigned to carbohydrates and polysaccharides by Munyendo et al. [72], Reis et al. [94], and Zhang et al. [105].

The statistically significant differences in integrated intensities (*p* < 0.05) across all major vibrational frequencies, as confirmed by one-way ANOVA, indicate a distinct biochemical composition between the two coffee species. This interpretation is supported by the Lambert-Beer law, which establishes a direct correlation between absorbance values and biofunctional metabolites levels [106,107,108,109,110,111].

This study is the first to provide a detailed FTIR characterization of robusta and excelsa coffees. The FTIR profiles obtained are consistent with those reported for the same species from other regions, although intensity differences may be attributed to agroclimatic conditions and post-harvest practices [95,112,113]. The less-studied excelsa shows a profile similar to that described in the literature, though potential compositional variations may be influenced by geographical origin [114]. The highly uncommon FTIR signature of excelsa underscores the significance of these findings. Excelsa exhibits a distinct lipid and carbohydrate signature that may impact its organoleptic and nutritional properties. Regionally, these findings position the Comoros as a producer of coffees with distinct biochemical profiles, offering opportunities for economic valorization within the international specialty coffee market [53], and pave the way for targeted metabolomic analyses.

## 5. Conclusions

This study provides the first comparative biochemical, ionomic, and vibrational spectroscopic characterisation of *Coffea canephora* (robusta) and *Coffea liberica* var. *dewevrei* (excelsa) cultivated under identical pedoclimatic conditions in the volcanic terroir of the Comoros Archipelago. The current results demonstrate that these two species, despite sharing the same growth environment, exhibit profoundly distinct and species-specific metabolic signatures. Comorian robusta is distinguished by significantly (*p* < 0.05) higher levels of total phenolics (121.8 ± 2.7 mg GAE/g DW), total flavonoids (29.4 ± 2.2 mg QE/g DW), caffeine (1.52% *w*/*w*), total soluble sugars (60.5 ± 3.4 mg GE/g DW), and DPPH radical-scavenging activity (65.0 ± 6.2 mM Trolox eq/g DW). Notably, the polyphenol content recorded here substantially exceeds literature values for the same species cultivated in Brazil (18–25 mg GAE/g DW) and Vietnam (80–100 mg GAE/g DW), underscoring the exceptional biochemical distinctiveness conferred by this specific volcanic terroir. These values position Comorian robusta as a high-value source of natural antioxidants and biofunctional metabolites. Conversely, excelsa displays a nutritionally unique and complementary mineral profile, with significantly higher concentrations of magnesium, calcium, sodium, zinc, and copper. This feature, reported here for the first time for this undercharacterised species, is directly linked to the basaltic, volcanic soils of the archipelago and highlights the terroir-driven mineral biofortification potential of excelsa. Crucially, FTIR-ATR spectroscopy independently confirms these interspecies differences through distinct vibrational fingerprints in the lipid, carbohydrate, and protein domains, with statistically significant differences observed across all major absorption bands (*p* < 0.05). This spectroscopic evidence provides a rapid, non-destructive diagnostic tool for species authentication and geographical traceability. Collectively, our findings position Comorian robusta as a potent antioxidant-rich coffee with notable stimulant properties, and excelsa as a mineral-dense, nutritionally balanced alternative with promising nutraceutical potential. These findings provide a strong scientific basis for the dual valorization of these underutilized genetic resources as specialized coffees, directly supporting biodiversity conservation and sustainable use in a climate-vulnerable region.

## Figures and Tables

**Table 1:** Biochemical, nutritional and mineral composition of robusta and excelsa cultivated in the Comoros: descriptive statistics and inter-species comparison.

Variable (Unit)	Group	Mean	SD	CV (%)	F-Statistic	*p*-Value	Tukey Sig.
**Total phenolics, total flavonoids, soluble sugars, tannins, antioxidant activity & caffeine**							
Total phenolics (TP) (mg GAE/g DW)	Excelsa	49.08	4.25	8.67%	620.64	1.50 × 10^−5^	Yes b
	Robusta	121.79	2.73	2.24%	620.64	1.50 × 10^−5^	Yes a
Total flavonoids (TF) (mg QE/g DW)	Excelsa	15.98	3.26	20.42%	35.03	0.0041	Yes b
	Robusta	29.43	2.20	7.46%	35.03	0.0041	Yes a
Total soluble sugars (TSS) (mg GE/g DW)	Excelsa	41.09	2.84	6.92%	57.93	0.0016	Yes b
	Robusta	60.47	3.37	5.58%	57.93	0.0016	Yes a
Hydrolysable tannins (HTs) (mg TAE/g DW)	Excelsa	8.88	2.79	31.48%	0.21	0.6721	No a
	Robusta	10.06	3.54	35.12%	0.21	0.6721	No a
DPPH antioxidant activity (mM Trolox eq/g DW)	Excelsa	41.74	5.29	12.67%	24.12	0.0080	Yes b
	Robusta	64.97	6.25	9.63%	24.12	0.0080	Yes a
Caffeine (%)	Excelsa	0.89	0.000	—%	1.58 × 10^37^	2.41 × 10^−56^	Yes a
	Robusta	1.52	0.000	—%	1.58 × 10^37^	2.41 × 10^−56^	Yes b
**Proximate composition: proteins & nitrogen**							
Proteins (%)	Excelsa	94.45	0.000	—%	5.25 × 10^33^	2.17 × 10^−49^	Yes a
	Robusta	95.48	0.000	—%	5.25 × 10^33^	2.17 × 10^−49^	Yes b
Total nitrogen (N) (%)	Excelsa	1.61	0.000	—%	2.54 × 10^36^	9.33 × 10^−55^	Yes a
	Robusta	1.86	0.000	—%	2.54 × 10^36^	9.33 × 10^−55^	Yes b
Macroelements (g/kg DW)							
Potassium (K) (g/kg DW)	Excelsa	31.20	0.000	—%	2.13 × 10^38^	1.32 × 10^−58^	Yes a
	Robusta	67.90	0.000	—%	2.13 × 10^38^	1.32 × 10^−58^	Yes b
Sodium (Na) (g/kg DW)	Excelsa	13.35	0.05	0.37%	7.94 × 10^9^	0.095	Yes a
	Robusta	7.60	0.10	1.32%	7.94 × 10^9^	0.095	Yes b
Calcium (Ca) (g/kg DW)	Excelsa	6.34	0.09	1.40%	28.72	0.0058	Yes a
	Robusta	6.00	0.06	1.04%	28.72	0.0058	Yes b
Magnesium (Mg) (g/kg DW)	Excelsa	12.99	0.07	0.50%	9.43 × 10^9^	0.067	Yes a
	Robusta	9.34	0.003	0.03%	9.43 × 10^9^	0.067	Yes b
Microelements (mg/kg DW)							
Iron (Fe) (mg/kg DW)	Excelsa	1.05	0.06	5.30%	17.61	0.0137	Yes b
	Robusta	1.20	0.03	2.13%	17.61	0.0137	Yes a
Zinc (Zn) (mg/kg DW)	Excelsa	0.33	0.007	2.14%	749.98	1.10 × 10^−5^	Yes a
	Robusta	0.13	0.010	7.46%	749.98	1.10 × 10^−5^	Yes b
Copper (Cu) (mg/kg DW)	Excelsa	0.34	0.003	0.88%	389.56	3.90 × 10^−5^	Yes a
	Robusta	0.30	0.003	0.84%	389.56	3.90 × 10^−5^	Yes b
Manganese (Mn) (mg/kg DW)	Excelsa	0.059	0.005	8.47%	30.62	0.0052	Yes b
	Robusta	0.079	0.004	4.46%	30.62	0.0052	Yes a

Different letters (a, b) within the same variable indicate statistically significant differences between groups (Tukey’s HSD post hoc test, *p* < 0.05).

**Table 2:** Assignment of major FTIR-ATR vibrational bands of robusta and excelsa and comparison with literature data.

Wavenumber (cm^−1^)	Functional Groups	Mode of Vibration	Intensity	Spectral Assignments	References
~3300	O-H	ν(O-H)	Broad	Hydroxyl stretching vibrations of phenolic acids (chlorogenic acid), polysaccharides and residual water	[70,71,78,79,80,81,82]
~2925	CH_2_	ν_as_(CH_2_)	Strong	Asymmetric stretching vibrations of lipid methyl groups and C–H bonds in caffeine molecules	[70,71,83,84]
~2850	CH_2_	ν_s_(CH_2_)	Strong	Symmetrical stretching of methylene groups in lipids and C–H bonds in caffeine molecules	[70,71,83,84]
~1740	C=O	ν(C=O)	Strong	Carboxylate ester stretching vibrations (triglycerides, lipid esters)	[83,85]
~1650	C=O, C=C	ν(C=O), ν(C=C)	Strong	Amide I (protein) and/or caffeine C=O/C=C stretching vibrations	[70,71,78,79,86,87,88]
~1550	N-H, C-N	δ(N-H) + ν(C-N)	Average	N-H bending + C-N stretching of amide II (proteins)	[70,71,78,89,90,91]
~1380	CH_2_, CH_3_	δ_as_(CH_3_), δ_s_(CH_2_)	Average	Asymmetric CH_3_ stretching and symmetric CH_2_ stretching vibrations of lipids	[70,71,89]
~1250	C-O-C	ν(C-O)	Strong	Stretching vibration of the C-O bond in secondary and primary hydroxyl groups of polysaccharides (cellulose, hemicellulose) and polyphenols	[90]
~1150	C-O	ν(C-O)	Average	C-O stretching (phenol-ether) of chlorogenic acids	[70,71,78,79,85,86,88,92,93]
~1020	C-O	ν(C-O)	Average	C-O stretching (primary alcohol) in quinic acid, carbohydrates and polysaccharides	[70,71,72,94]

ν = stretching (elongation); δ = deformation (scissoring, bending); s = symmetric; as = asymmetric.

## Data Availability

The original contributions presented in the study are included in the article. Further inquiries can be directed to the corresponding author.
